# Address to the Veterinary Profession of the State of New Jersey1Delivered January 11, 1900, on the occasion of the veterinary societies of the State merging with the Veterinary Medical Association of New Jersey.

**Published:** 1900-03

**Authors:** William Herbert Lowe

**Affiliations:** Paterson, N. J.


### ADDRESS TO THE VETERINARY PROFESSION OF THE STATE OF NEW JERSEY.[1]

By William Herbert Lowe, D.V.S.,
PATERSON, N. J.

Now, in the dawn of the twentieth century, we witness the beginning of a new epoch in the history of the progress of veterinary science in the State of New Jersey. It is not necessary to dwell upon the early struggles of individuals and of societies for existence and life during the era now at a close, but let us pause for a moment.

Heretofore the profession of our State has been divided up into various factions and societies, each trying to accomplish what it could. To-day these societies are merged and amalgamated into one strong State organization—the Veterinary Medical Association of New Jersey—truly representative of the profession of the State. This means one strong State organization instead of two or three societies more dead than alive; this means unity, harmony, and strength. Now we may expect to see a steady, healthy growth in veterinary science and art, with a consequent benefit to man and beast.

To greet the veterinary societies of the State as an amalgamated association, and to welcome the officers and members of each, is a privilege I esteem very highly.

This, I believe, is not only the largest, but the most earnest and truly representative gathering of members of the veterinary profession that has ever assembled within the confines of the State, which in itself speaks louder than language that the profession is stirred as never before in her history.

It has been said that I was, like the commercial men of the day, organizing a "veterinary trust." I think veterinarians have enough trusting to do without any organizing. However, I am willing for it to be called a "veterinary trust" so long as we can have unity, harmony, and strength. Three months ago some of the oldest and wisest men in the profession shook their heads and said that it was impossible to unite and organize the veterinary factions in this State. What seemed impossible so short a time ago is now an accomplished fact.

It was thought wise to build upon the old foundation laid many years ago. I refer to the Veterinary Medical Association of New

[1] Delivered January 11, 1900, on the occasion of the veterinary societies of the State merging with the Veterinary Medical Association of New Jersey.

Jersey, organized in the year 1884 and incorporated in 1885 under an act of the Legislature for the promotion of veterinary science and art. This act was repealed in 1899, so no more veterinary societies can be incorporated under it, and inasmuch as the other two incorporated societies have to-day merged with the original association, that leaves the Veterinary Medical Association of New Jersey the only chartered veterinary society in the State and does away with the provision for any more societies being incorporated, which is a good thing.

Some of the members of the old association did not have the advantage of collegiate and scientific training of the veterinarian of to-day, but many of those men deserve a great deal of credit for what they did accomplish with the limited means at their command. I want to say to the profession here assembled that I have a great deal of respect for many of these men. I have known some of them, such as the lamented Dr. Dustan, of Morristown, who had qualities of mind and heart that more than compensated for lack of collegiate training.

The large number of candidates for membership at this meeting is phenomenal. I have had the honor of presenting the names of fifty-two practitioners for membership, most of whom have already paid their initiation fees. Fifty-two new members certainly ought to throw new life into the various branches of the association's work. I predict that the Veterinary Medical Association of New Jersey can be made one of the, if not the, strongest and best veterinary society in this whole United States. There is plenty of good timber in New Jersey. It will not do to let Dr. Hoskins have all his own way over in Pennsylvania.

I would like to see this association inclusive rather than exclusive. Of course, all will not have equal education or equal ability. This is not found even among the graduates of any particular college or university, but all the more reason for and importance of society attachment.

The advantages of a proper State organization are many. First, it gives practitioners an opportunity of getting acquainted with each other, and tends to create a fraternal feeling. Of course, if a man has not character he will do mean and contemptible things, whether he is in the society or out. In the society, however, he can be disciplined and brought under moral restraint; whereas, if outside nothing can be done with him unless he directly violates a law of his city or of the State.

In the second place, the State Association is of great importance in the promotion of scientific advancement, which every truly professional man is interested in. There is not a veterinarian in this gathering but who knows certain things that would be of advantage to some other veterinarian; and then, again, this some other veterinarian knows things that his brother does not know. There should be an exchange of experiences, opinions, etc. Cases should be reported; not those only that recover, but more particularly those that do not recover. The practitioner's opinion as to why cases did not recover should be given. Post-mortem examinations should be the rule, and not the exception, if the profession is to advance in pathology and in the scientific treatment of disease.

The examination of horses as to soundness is a subject that this association could profitably take up, and one that every practitioner would take an interest in and one that every veterinarian would profit by—there seems to be such a difference of opinion among practitioners as to what constitutes soundness and what constitutes unsoundness.

We have here with us a veterinarian who has given a great deal of special study and thought to normal and pathological horseshoeing, who could instruct and interest us. I refer to our friend Dr. McDonough. There are others who perhaps could add to Dr. McDonough's experience, and so on.

Other members would be better qualified to discuss dental and general surgical manipulations and operations. In this connection I would like to say I can see no good reason why we cannot have surgical clinics at our meetings; they are already becoming quite a feature at the meetings of the National Association.

Sanitary medicine, State medicine, veterinary inspection of the meat and milk food-supply, and a proper veterinary inspection of the animals that produce the supply are questions that concern the health as well as the wealth of the people of the State, and it is a body of this kind that is essentially qualified to study and deal with the many complex problems that are connected with this important subject. I could go on indefinitely indicating scientific work that we as an organization must take account of, but I must pass on to another advantage that a strong State organization is to the profession and to the public alike. I refer to veterinary legislation. We can only expect to get such legislation as the profession should have by being united and by earnest and persistent effort of the association as a body as of its members individually. Our veterinary law passed in 1899 will have to be amended. This can be done if we stand together. We should have a State Board of Veterinary Medical Examiners in this State similar to the boards in Pennsylvania and New York. It is a notorious fact that men who cannot pass before these boards come over to New Jersey and set themselves up as veterinarians. Is the profession going to stand quietly by and allow New Jersey to become the "dumping-ground" of professional incompetents? I think not.

There is an illimitable amount of professional work for the veterinarian who has the true interests of the profession at heart. The field is ever broadening; the extent to which the health and wealth of the people depend upon the application of veterinary science is becoming more appreciated day by day. I see a great future for the Veterinary Medical Association of New Jersey as now constituted. May she ever be true to herself and to the interests of the people of our beloved State.

